# The Effect of Vitamin D Supplementation in Pregnant Women with Overweight and Obesity: A Randomised Controlled Trial

**DOI:** 10.3390/nu16010146

**Published:** 2023-12-31

**Authors:** Chee Wai Ku, Angeline Jia Wen Lee, Benjarat Oh, Celeste Hong Fei Lim, Ting Yu Chang, Fabian Yap, Jerry Kok Yen Chan, See Ling Loy

**Affiliations:** 1 Department of Reproductive Medicine, KK Women’s and Children’s Hospital, 100 Bukit Timah Road, Singapore 229899, Singapore; gmskcw@nus.edu.sg (C.W.K.); benjarat.oh@kkh.com.sg (B.O.); jerrychan@duke-nus.edu.sg (J.K.Y.C.); 2 Duke-NUS Medical School, 8 College Road, Singapore 169857, Singapore; fabian.yap.k.p@singhealth.com.sg; 3 Lee Kong Chian School of Medicine, Nanyang Technological University, 59 Nanyang Drive, Experimental Medicine Building, Singapore 636921, Singapore; ange0056@e.ntu.edu.sg; 4 Yong Loo Lin School of Medicine, National University of Singapore, 10 Medical Drive, Singapore 117597, Singapore; celestelhf@gmail.com (C.H.F.L.); tingyu452@gmail.com (T.Y.C.); 5 Endocrinology Service, Department of Paediatrics, KK Women’s and Children’s Hospital, 100 Bukit Timah Road, Singapore 229899, Singapore

**Keywords:** vitamin D deficiency, vitamin D supplement, obesity, pregnancy, lipid profile, 25OHD

## Abstract

The impact of vitamin D supplementation on 25-hydroxyvitamin D (25OHD) levels, metabolic status, and pregnancy outcomes in pregnant women with overweight and obesity (OW/OB) is uncertain. This study aimed to examine whether administrating 800 IU of vitamin D3 orally would improve maternal serum 25OHD levels, lipid profile, and pregnancy outcomes compared to 400 IU. This was a two-arm, parallel, non-blinded randomised controlled trial involving 274 pregnant women recruited from KK Women’s and Children’s Hospital, with a body mass index of ≥25 kg/m^2^ within 16 weeks gestation. The participants were randomly assigned to receive 800 IU/day (intervention group) or 400 IU/day (control group) of oral vitamin D3 supplements. The primary outcomes were maternal serum 25OHD and lipid levels at 24–28 weeks gestation. The secondary outcomes included maternal and birth outcomes. Compared with controls (*n =* 119), the intervention group (*n =* 112) exhibited higher 25OHD levels at 24–28 weeks gestation (adjusted mean difference 6.52 nmol/L; 95% confidence interval 2.74, 10.31). More women in the intervention group achieved sufficient 25OHD levels (77.7% vs. 55.5%; *p* < 0.001). No differences were observed in lipid profiles or maternal or birth outcomes between the groups. An additional 400 IU of oral vitamin D3 supplementation increased serum 25OHD levels but did not impact lipid profiles or pregnancy outcomes.

## 1. Introduction

Vitamin D, an essential nutrient, is primarily produced through the conversion of 7-dehydrocholesterol in the skin upon exposure to ultraviolet B radiation. Aside from its well-established role in calcium homeostasis, vitamin D regulates numerous cellular processes [1]. Insufficient vitamin D levels have been linked to multiple adverse health outcomes, including low bone mineral density, autoimmune diseases, and various forms of cancers [2]. With approximately one billion individuals globally experiencing insufficient vitamin D levels, this has emerged as a significant worldwide public health concern [2]. Pregnant women, especially, face a heightened risk of vitamin D insufficiency [3]. Despite Singapore having a tropical climate year-round, many pregnant women have low serum vitamin D levels, as indicated by circulating 25-hydroxyvitamin D (25OHD) [4]. Notably, a lack of 25OHD is associated with unfavourable pregnancy outcomes, including pre-eclampsia, preterm birth, and low birth weight [5,6,7,8,9]. Vitamin D deficiency during pregnancy is also associated with various adverse health outcomes during childhood. Insufficient 25OHD levels may contribute to seizures and dilated cardiomyopathy caused by hypocalcaemia [10]. Furthermore, it is associated with an increased incidence of acute lower respiratory tract infections and recurrent wheezing in the first five years of life [11]. Several studies have also shown a heightened risk of allergic diseases due to inadequate 25OHD levels in mothers during pregnancy [12].

However, the existing literature revealed conflicting results regarding the impact of vitamin D supplementation during pregnancy. While some studies suggest that antenatal vitamin D supplementation can improve serum 25OHD levels and reduce the risks of the aforementioned complications [13,14,15,16], others have failed to show a positive effect [17,18]. This ambiguity is particularly pronounced in pregnant women with overweight and obesity (OW/OB), who face an elevated risk of pregnancy complications and are more prone to vitamin D insufficiency due to sequestration of this fat-soluble vitamin within adipocyte lipid droplets [19]. This increased volume of distribution of vitamin D impacts the effect of vitamin D supplementation [20]. Although vitamin D is considered an antiatherogenic agent due to its potential beneficial effects on lipid metabolism and its anti-inflammatory potency [21,22], its effects on the metabolic status of OW/OB pregnant women remain uncertain. Previous studies have indicated an association between vitamin D deficiency and an unfavourable lipid profile [15,23,24,25]. Moreover, there is evidence suggesting that vitamin D supplementation could improve metabolic profiles in women with gestational diabetes [26]. In addition, the appropriate dosage of vitamin D supplementation during pregnancy remains a subject of contention. In Singapore, the current standard of care involves supplementing pregnant women with 400 IU/day of vitamin D through antenatal multivitamins. Meta-analyses of randomised controlled trials suggest that vitamin D supplementation during pregnancy, with doses ≤ 2000 IU daily, lowers the risk of infants being small for gestational age and enhances growth during infancy without increased risk of foetal or neonatal mortality or congenital abnormalities [27]. Pilz et al. [7] propose a safe range of 800–1000 IU/day during preconception or early pregnancy to ensure an adequate supply of vitamin D for the foetus or infant. This is supported by a randomised trial in pregnant women, which reported that supplementation of 800 IU/day since early pregnancy could maintain maternal and foetal 25OHD at sufficient levels [19].

Nearly one-third of women in Singapore are affected by OW/OB (body mass index (BMI) ≥ 25 kg/m^2^) during pregnancy [4]. We conducted a randomised trial to investigate whether intervention with an 800 IU oral vitamin D3 supplement (comprising 400 IU of vitamin D3 standalone supplement and 400 IU of vitamin D3 standard prenatal multivitamin supplement) compared to a 400 IU of vitamin D3 standard prenatal multivitamin, taken from early pregnancy until delivery, would lead to improved maternal serum 25OHD levels, lipid profiles, and pregnancy outcomes in women with OW/OB during pregnancy. We hypothesised that, among pregnant women with OW/OB, those receiving the 800 IU vitamin D3 supplement would exhibit higher serum 25OHD levels, improved lipid profiles, and better pregnancy outcomes compared to women receiving the 400 IU vitamin D3 supplement.

## 2. Materials and Methods

### 2.1. Study Design

This study employed a two-arm, parallel, non-blinded randomised controlled trial design, registered under NCT 04841265. Pregnant women were recruited from KK Women’s and Children’s Hospital between June 2021 and November 2022. This research adhered to the principles outlined in the Declaration of Helsinki and received ethics approval from the SingHealth Centralised Institutional Review Board (reference 2021/2055).

### 2.2. Participants

Participants were eligible if they were aged 21 to 45 years, with a pre-pregnancy BMI of ≥25 kg/m^2^ and were within 16 weeks of gestation. Individuals with specific current or past medical histories, as identified at recruitment, were excluded. These included hypo/hyperparathyroidism, hypercalciuria, hypercalcemia, osteomalacia, liver dysfunction, tuberculosis, renal disease, and sarcoidosis. Individuals with multiple pregnancies, pre-existing diabetes mellitus, diagnosed gestational diabetes mellitus (GDM) or gestational hypertension at recruitment, chronic hypertension, or use of lipid-lowering medication were also excluded. Additionally, participants were withdrawn in cases of miscarriage, ectopic pregnancy, or adverse reactions. All participants provided written informed consent.

### 2.3. Data Collection

Participants completed baseline investigations during the antenatal visit between 10 and 16 weeks gestation. Detailed questionnaires were administered in the clinic by the research staff. Participants provided information on socio-demographics, dietary intake, and lifestyle factors including ethnicity, education, employment status, sun exposure, sunscreen use, dietary sources rich in vitamin D and/or calcium, consumption of supplements containing vitamin D, calcium, and/or omega-3 fatty acids, smoking exposure, sedentary time, and physical activity levels (determined using International Physical Activity Questionnaire-Short Form [28] to derive the metabolic equivalent of task score (MET-min/week). The intensity of physical activity was subsequently categorised as inactive (<600 MET-min/week), minimally active (600 to <3000 MET-min/week), and highly active (≥3000 MET-min/week). Research staff measured weight (kg) and height (m) of participants in the clinic, with their BMI (kg/m^2^) calculated by the Avalanche Mechrotonics B1000M BMI machine, Singapore. At the end of the baseline visit, blood samples were collected to assess fasting serum 25OHD levels and lipid profile.

At 24–28 weeks gestation, research staff performed follow-up assessments on diet and lifestyle through questionnaire administration. Participants underwent a 3-point (0, 1 and 2 h) 75 g oral glucose tolerance test in the morning after an overnight fast of 8 to 10 h, following standard clinical protocols. After delivery, data on obstetric complications, delivery, and birth outcomes were retrieved from hospital case notes.

### 2.4. Interventions

Participants in the control group received standard prenatal multivitamin supplement tablets, “Obimin”, which contained 400 IU of vitamin D3, along with folic acid, vitamin B1, B2, B6, B12, and other minerals. Participants in the intervention group were additionally provided with an oral-dissolving vitamin-D3-only supplement, contributing an extra 400 IU, alongside the multivitamin tablet (Obimin). Participants in the control group received 400 IU of vitamin D3 daily, while those in the intervention group received 800 IU of vitamin D3 daily. The supplementation was continued from the baseline visit until delivery without any dosage alterations midway through the study. Staff monitored compliance through tablet counts. Participants were advised to maintain their levels of physical activity and dietary intake, and refrain from taking any other supplements containing vitamin D throughout the study period.

### 2.5. Outcomes

Primary outcomes included maternal serum 25OHD and lipid levels. Serum 25OHD levels were assessed using electrochemiluminescence technology for immunoassay (Roche Cobas E411, Switzerland) and classified as sufficient (25OHD ≥ 50 nmol/L), insufficient (25OHD 25 to < 50 nmol/L), and deficient (25OHD < 25 nmol/L). Currently, there are no standardised criteria for 25OHD levels. The UK Scientific Advisory Committee on Nutrition has proposed that a serum 25OHD level < 25 nmol/L indicates a concentration at which the risk of poor musculoskeletal health is increased at a population level and is therefore considered indicative of vitamin D deficiency [29]. Moreover, despite varying evidence regarding what constitutes sufficient 25OHD concentrations, numerous studies have concluded that a 25OHD level > 50 nmol/L is generally considered adequate by most experts [30]. Currently, there is also no evidence supporting the use of a different criterion for pregnant adults [31]. Total cholesterol levels were assessed by the Abbott Alinity c Cholesterol Reagent (Cholesterol oxidase, peroxidase) kit, Germany; triglycerides levels were assessed by the Abbott Alinity c Triglyceride Reagent (Lipase, Glycerol kinase, GPO-PAP) kit, Germany; high-density lipoprotein cholesterol (HDL-C) levels were assessed by the Abbott Alinity c Ultra HDL Reagent (Accelerator, Selective detergent) kit, Germany; low-density lipoprotein cholesterol (LDL-C) levels were determined by subtracting HDL-C (mmol/L) and triglyceride/2.2 (mmol/L) from the total cholesterol (mmol/L) [32].

Secondary maternal outcomes included fasting glucose, 1 h post-load glucose, 2 h post-load glucose, GDM, gestational hypertension, pre-eclampsia, caesarean section, and total gestational weight gain. Plasma glucose levels were assessed by the Abbott Alinity c glucose enzymatic (Hexokinase) assay, Germany. GDM was diagnosed based on the International Association of Diabetes and Pregnancy Study Groups criteria [33]. Gestational hypertension was defined as systolic blood pressure of ≥140 mm Hg and/or diastolic blood pressure of ≥90 mm Hg [34]. Pre-eclampsia was defined as new-onset gestational hypertension with proteinuria (≥0.3 g protein in a 24 h urine specimen) [34]. Gestational weight gain was defined by the 2009 Institute of Medicine Guidelines [35]. Secondary birth outcomes included birth weight, birth length, head circumference at birth, preterm birth (<37 completed gestation weeks), low birth weight (<2500 g), and admission to special care during the neonatal period.

### 2.6. Sample Size

We calculated sample sizes based on the hypotheses of achieving (1) higher serum 25OHD; and (2) lower LDL levels in the intervention group compared to the control group. Assuming medium effect sizes of 0.4 standard deviation (SD) differences in serum 25OHD (with a SD of 15 nmol/L) and LDL levels (with a SD of 0.7 mmol/L) between the intervention and control groups, we required a sample size of 113 per group, ensuring at least 80% power and with two-sided 5% type 1 error rate. Considering 25% dropout rate, we aimed to recruit a total of 300 participants, with 150 per group.

### 2.7. Randomisation

An independent investigator, not involved in participant recruitment or data collection, performed random assignments using a computer-generated randomisation code. Participants were randomly assigned to either the intervention or control group in a 1:1 ratio.

### 2.8. Statistical Analysis

We employed standard summary statistics to describe baseline characteristics. Categorical variables were summarised in frequency and percentage; continuous variables were reported in mean and SD, as well as median and interquartile range, depending on their distributions. We compared characteristics between control and intervention groups using the Pearson’s chi-squared test for categorical variables and the independent *t*-test for continuous variables.

We used a modified intention-to-treat approach, including all randomly assigned participants with available outcome data. We excluded participants withdrawn before the 24–28 weeks gestation follow-up. For primary outcomes, mean differences and corresponding 95% confidence intervals (CIs) between groups were estimated and adjusted for baseline values and intervention duration through the analysis of covariance test. For secondary outcomes, we compared group differences using the independent *t*-test or Pearson’s chi-squared test, as appropriate. We performed additional analyses by stratifying participants into overweight and obese categories, restricting the analysis to participants with persistent 25OHD insufficiency, or those with at least 80% compliance [36,37]. We used a two-sided significance level of 5% for pre-specified primary and secondary outcomes without adjustment for multiple comparisons. We conducted statistical analysis using SPSS statistical software (version 20; IBM Corp., Armonk, NY, USA).

## 3. Results

**Figure 1:**
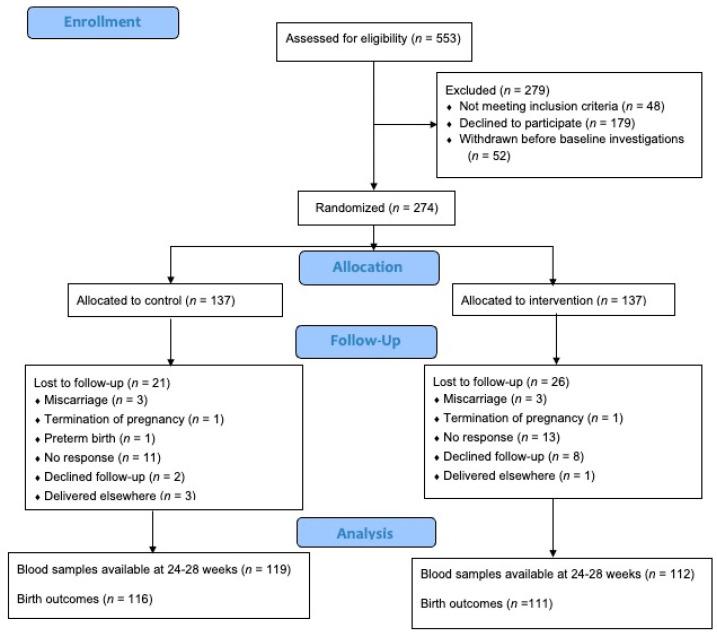
Flow diagram of participants.

Out of the 553 pregnant women who were assessed for eligibility, 274 were included and randomised (Figure 1). Of these, 231 (84.3%) participants completed the follow-up at 24–28 weeks gestation and 227 (82.8%) remained in the trial until delivery. The mean age and BMI of the participants were 30.58 years (SD 4.46) and 30.00 kg/m^2^ (SD 4.25), respectively. The majority (*n =* 157, 57.3%) of the participants were of Malay ethnicity. The mean plasma 25OHD level at baseline was 39.44 mmol/L (SD 14.71), with more than half (*n =* 174, 63.5%) displaying insufficient levels. No significant differences in baseline characteristics, plasma 25OHD, and lipid levels were observed between the control and intervention groups (all *p*-values > 0.05) (Table 1).

Table 2 presents the outcome measurements at 24–28 weeks gestation. The participants from the intervention group showed a higher level of plasma 25OHD (61.45 nmol/L; SD 16.74) than those from the control group (53.46 nmol/L; SD 16.20). The increase in 25OHD from baseline until 24–28 weeks gestation for the intervention group was greater than the control group (21.52 nmol/L vs. 14.52 nmol/L)**.** After adjusting for baseline 25OHD and intervention duration, the estimated difference in 25OHD between groups was 6.52 nmol/L (95% CI 2.74, 10.30). More participants from the intervention group achieved sufficient levels of vitamin D with 25OHD ≥ 50 nmol/L (*n =* 87, 77.7%) than those from the control group (*n =* 66, 55.5%). Following supplementation, there were no significant differences in lipid profile, OGTT, and gestational diabetes between the control and intervention groups (Table 2). When we restricted the analysis to a subgroup of women who remained 25OHD-insufficient from baseline until 24–28 weeks gestation (*n =* 54), no significant differences in lipid and glycaemic measures were observed between groups, except for HDL-C levels, which were lower in the intervention group (1.53 mmol/L; SD 0.23) than in the controls (1.82 mmol/L; SD 0.39), *p* = 0.012 (Table S1). For maternal and birth outcomes at delivery, no significant differences were observed between the control and intervention groups (Table 3).

Furthermore, as the study progressed until 24–28 weeks, the control group exhibited calcium intake of 370.44 mg/day (25th–75th centiles: 195.68–567.12), while the intervention group showed an intake of 345.55 mg/day (25th–75th centiles: 100.17–585.45). However, no significant difference between the groups was identified at this stage (*p* = 0.293). No associations were detected between dietary calcium intake and 25OHD levels at both baseline (*p* = 0.552) and 24–28 weeks gestation (*p* = 0.371).

After classifying the participants by overweight and obesity status, the results for plasma 25OHD, lipid, and glycaemic measures remained consistent (Table S2). However, the participants with 25OHD deficiency displayed a trend toward lower HDL-C than those with sufficient levels (*p* = 0.058) (Table 4). In terms of birth outcomes, infants born to women with obesity who received the intervention had a lower incidence of low birth weight (3.9% vs. 17.0%, *p* = 0.032) and longer birth length (49.06 cm vs. 48.06 cm, *p* = 0.024) compared to those born to women with obesity in the control group (Table S3).

The overall compliance rate with supplementation was 89.9%. When analysing a subset of participants with a compliance rate of 80% and above, similar findings were observed for plasma 25OHD, lipid, and glycaemic measures (Table S4). However, within this subgroup of women, those receiving the intervention were more likely to deliver infants with a longer birth length (48.82 cm vs. 48.21 cm, *p* = 0.042) and a greater head circumference (34.05 cm vs. 33.65 cm, *p* = 0.042) than the controls (Table S5). No participants discontinued supplements or withdrew from the trial due to adverse events (Table S6).

## 4. Discussion

In pregnant women with OW/OB, administering 800 IU of oral vitamin D3 supplement from early pregnancy significantly increased serum 25OHD levels and the proportion of women achieving sufficient 25OHD compared to the control group receiving 400 IU of vitamin D3 supplement at 24–28 weeks gestation. This higher supplementation dose effectively prevented vitamin D deficiency, as indicated by serum 25OHD < 25 nmol/L in this population. However, the additional 400 IU of vitamin D3 in the intervention had no discernible impact on lipid profiles and glycaemic measures, although a marginal effect on improving foetal growth was observed specifically in women who complied with the intervention or those with obesity.

Our findings are aligned with a randomised clinical trial involving pregnant women with OW/OB, which reported a significant difference of 8.7 nmol/L and 10.3 nmol/L in 25OHD levels between groups receiving 400 IU/day and 800 IU/day of supplementation, respectively, in women with OW/OB [19]. In another clinical trial, an increased dosage of 1000 IU/day supplementation resulted in 90% of pregnant women achieving a sufficient 25OHD level of 50 nmol/L [38]. Additionally, our study observed a 21.5 nmol/L increase in 25OHD levels with 800 IU/day of supplementation, consistent with several randomised clinical trials that showed that every 40 IU of vitamin D3 supplementation resulted in a 0.6–1.2 nmol/L elevation in 25OHD levels [39]. These findings collectively suggest that the current standard of care of 400 IU/day of vitamin D3 supplement is inadequate in preventing 25OHD deficiency during pregnancy, particularly in women with OW/OB who exhibit reduced 25OHD response to vitamin D3 supplementation due to adipose tissue sequestration [40]. Therefore, a higher supplementation dose, preferably at least 800 IU/day, may be imperative to achieve optimal vitamin D status during pregnancy.

The impact of vitamin D3 supplementation on lipid metabolism during pregnancy remains a complex and debated topic. Despite the known role of vitamin D in reducing liver triglyceride synthesis and cholesterol conversion to bile acids [41], our study, employing a daily dosage of 800 IU, did not yield significant effects on lipid profile. This contrasts with some studies advocating for supplementation due to the association between vitamin D insufficiency and unfavourable lipid profiles [15,23,24,25]. However, the lack of impact is consistent with findings from studies administering doses of 1600–2000 IU/day [15,25]. Intriguingly, within our subgroup analysis, women with persistent 25OHD insufficiency demonstrated a nuanced effect, showcasing lower HDL-C levels in the intervention group compared to the control counterparts, likely due to metabolite interactions, such as with leptin [42]. An increase in vitamin D level following supplementation has been shown to increase leptin levels [42], which has been reported to be inversely correlated with HDL-C levels [43]. Hence, further investigation into potential metabolite interactions may explain the lower HDL-C levels in our intervention group. Studies utilising substantially higher doses, such as Huang et al.’s [26] administration of 40,000 IU of vitamin D3 along with omega fatty acids, demonstrated significant improvements in lipid profiles. Nonetheless, the absence of 25OHD level measurements in this study complicates the determination of the precise threshold for lipid changes. Regardless, this raises the possibility that doses beyond 800 IU/day may be necessary to effectively impact lipid metabolism, especially during pregnancy when hormonal changes lead to undesirable lipid measurements [44]. The baseline lipid profiles of our participants could partially explain the lack of significant results [45] as they presented relatively favourable profiles [46]. Previous studies showing lipid improvements through supplementation often involved participants with poorer baseline lipid levels [18,26], contrasting with studies reporting no improvements, which involved participants with initially favourable lipid levels, mirroring our findings [15,25]. Furthermore, the modest changes typically observed in lipid profiles following vitamin D3 supplementation [45], coupled with the increased sequestration of fat-soluble vitamin D in adipose tissues of women with OW/OB [40], present additional factors. Although the observed increase in 25OHD levels aligns with expectations [39], the challenge lies in demonstrating corresponding improvements in lipid levels. Altogether, unravelling the intricate relationship between vitamin D3 supplementation, 25OHD levels, and lipid profiles during pregnancy requires consideration of dose–response dynamics, participant baseline characteristics, and the interplay of various metabolites. Future studies exploring higher yet safe doses, metabolite interactions, and the influence of baseline characteristics on supplementation outcomes could provide valuable insights into optimizing maternal metabolic health.

Vitamin D is crucial for maintaining optimal maternal and foetal health during pregnancy. Numerous studies have demonstrated that low 25OHD levels are associated with adverse effects on maternal health, including an elevated risk of gestational diabetes, pre-eclampsia, caesarean section, preterm delivery, miscarriages, and postpartum depression [47]. Adverse foetal outcomes such as low birth weight, small head circumference, short body length, and foetal abnormalities are also known sequelae of poor maternal 25OHD levels [48]. Vitamin D facilitates the intestinal absorption of calcium, crucial for foetal skeleton development throughout pregnancy; inadequate maternal 25OHD levels are associated with poor musculoskeletal growth [49]. Moreover, insufficient calcium levels resulting from vitamin D deficiency can contribute to foetal heart problems since calcium is a critical ion for cardiac function [50]. Vitamin D also plays a neuroprotective role through the promotion of neurotropin release, facilitating optimal neurodevelopment [51]. Based on this evidence, it is postulated that vitamin D3 supplementation has the potential to prevent such complications. While the increase in 25OHD levels may appear modest (8.00 nmol/L or 3.20 ng/mL), it effectively elevated the proportion of women reaching sufficient levels (≥50 nmol/L) while preventing maternal deficiency (<25 nmol/L). This observed rise in 25OHD levels aligns with a previous investigation that employed similar supplementation doses in pregnant women with OW/OB. This earlier study demonstrated positive foetal outcomes, including elevated umbilical 25OHD, increased birth weight, and head circumference [19]. While our study did not reveal significant overall effects on specified pregnancy complications and birth outcomes, the marginal impact on birth size among women with high compliance or those with obesity conveys two important messages: (1) the significance of supplementation adherence for optimising foetal growth; and (2) the potential for a more responsive effect to the supplements in women with obesity compared to those with overweight status. We observed no differences in compliance rate and baseline 25OHD between women with OW/OB (data not shown). Nevertheless, achieving substantial improvements in maternal and foetal outcomes may require considering a higher dose of vitamin D3 supplementation, especially in women who start pregnancy with low 25OHD levels [19]. Additionally, the absence of significant findings in our study could be attributed to its limited statistical power in assessing the impact of vitamin D3 supplementation on secondary maternal and birth outcomes. This underscores the need for caution in drawing definitive conclusions.

Taken together, our study showed that over three-quarters of pregnant women with OW/OB had serum 25OHD levels below 50 nmol/L in early pregnancy. Consuming a daily dose of 800 IU of vitamin D3 supplementation effectively increased the proportion of women achieving sufficient 25OHD levels, surpassing 50 nmol/L in late-mid pregnancy, with no adverse events directly related to the supplementation reported. Given the high prevalence of vitamin D insufficiency in our population of pregnant women with OW/OB, implementing universal supplementation with a minimum dose of 800 IU/day of vitamin D3 may be cost-effective [19]. Additionally, considering the high prevalence of vitamin D insufficiency even among pregnant women without OW/OB in Singapore, future research could explore the feasibility and cost-effectiveness of a screen-and-treat approach for this population. This has potential long-term implications for maternal and child health, including improved musculoskeletal and emotional health in mothers [47,49], as well as enhanced cardiovascular, respiratory, and neurodevelopmental outcomes in offspring [11,50,51]. Further research is needed to explore sustained health advantages and emphasise the importance of maintaining optimal vitamin D status throughout the prenatal period.

Our study stands out as one of the rare randomised controlled trials investigating the effects of oral vitamin D3 supplementation on serum 25OHD and metabolic biomarkers specifically in pregnant women with OW/OB. These findings offer crucial insights into the appropriate vitamin D supplementation doses needed for this population, particularly in an Asian context. The early initiation and sustained administration of vitamin D3 supplementation until delivery, along with high participant compliance, enhanced the reliability of our findings. However, our study faced limitations in adequately powering the analysis of secondary outcomes, warranting a specifically designed and powered study focused on maternal and birth outcomes in response to varied vitamin D3 supplementation dosages. Moreover, we did not explore the use of higher supplementation doses that have shown significant effects in other studies. While the study excluded individuals using lipid-lowering medications and those with chronic hypertension taking antihypertensive drugs, individuals taking other medications like benzodiazepines, antidepressants, and proton pump inhibitors, which have been shown to affect vitamin D status, were not excluded [45]. Although these factors may have contributed to the absence of clinical changes in lipid levels, as well as maternal and foetal outcomes, this is deemed less likely considering the significant and expected increase in 25OHD levels in our study. Additionally, women who conceived through in vitro fertilization were not excluded, and modes of conception were not collected. This introduces potential heterogeneity due to differences in risk factors, characteristics, and underlying health conditions in comparison to naturally conceived pregnancies. Drawing clear conclusions about the impact of vitamin D supplementation on pregnancy outcomes within a more homogeneous group is therefore challenging.

Moreover, there are some limitations in the generalisability of our findings. While our study offers a distinctive perspective on vitamin D deficiency in the Asian context, our unique geographical location and ethnic composition may restrict the broader application of our study’s findings. Situated just 1° north of the equator, Singapore has a tropical rainforest climate characterised by the absence of distinct seasons. This results in no seasonal variation in UV exposure, which is not observed in many other countries [52]. This geographical uniqueness poses a potential limitation when extrapolating our findings, especially to populations experiencing variations in sun exposure due to seasonal changes, considering the effect of sunlight on vitamin D synthesis [53]. However, despite the availability of sunlight, numerous studies have identified that vitamin D deficiency is more prevalent in Asians [54,55]. This can be attributed to factors such as darker skin pigmentation, which adversely affects the speed of vitamin D synthesis, as well as prevalent sun-avoidance behaviours and a dietary pattern lacking in foods high in vitamin D [54,56].

## 5. Conclusions

In conclusion, 800 IU/day of vitamin D3 supplementation effectively increased the 25OHD levels and improved the vitamin D sufficiency status in pregnant women with OW/OB in pregnancy. However, we found no effect on lipid profiles or pregnancy outcomes. Larger trials with varying higher vitamin D3 supplement doses and increased sample sizes are warranted to examine the impact on maternal and birth outcomes in this population. In subsequent studies, it would also be valuable to delve deeper into the effects of vitamin D3 supplementation on long-term maternal and foetal outcomes beyond delivery timepoint.

## Figures and Tables

**Table 1:** Comparisons of maternal baseline characteristics between trial groups.

Variable	Total (*n =* 274)	Control (*n =* 137)	Intervention (*n =* 137)	*p*
Age, mean (SD), years	30.58 (4.46)	30.67 (4.22)	30.50 (4.69)	0.746
Gestation age at recruitment, mean (SD), weeks	11.78 (2.11)	11.89 (2.21)	11.66 (2.01)	0.367
Body mass index at recruitment, mean (SD), kg/m^2^	30.00 (4.25)	29.71 (4.14)	30.3 (4.35)	0.274
Ethnicity, *n* (%)				0.312
Chinese	72 (26.3)	38 (27.7)	34 (24.8)	
Malay	157 (57.3)	73 (53.3)	84 (61.3)	
Indian	26 (9.5)	13 (9.5)	13 (9.5)	
Others	19 (6.9)	13 (9.5)	6 (4.4)	
Education, mean (SD), years	14.0 (3.52)	14.1 (3.64)	13.9 (3.40)	0.601
Employment status, *n* (%)				0.559
Unemployed	60 (21.9)	32 (23.4)	28 (20.4)	
Employed	214 (78.1)	105 (76.6)	109 (79.6)	
Sun exposure 11 a.m.–3 p.m., *n* (%)				0.460
<20 min/ day	164 (59.9)	79 (57.7)	85 (62.0)	
≥20 min/ day	110 (40.1)	58 (42.3)	52 (38.0)	
Sunscreen usage, *n* (%)				0.390
None	151 (55.1)	76 (55.5)	75 (54.7)	
1–5 times/week	67 (24.5)	37 (27.0)	30 (21.9)	
6–7 times/week	56 (20.4)	24 (17.5)	32 (23.4)	
Consumption of supplements containing Vitamin D or calcium, *n* (%)				0.889
No	205 (74.8)	103 (75.2)	102 (74.5)	
Yes	69 (25.2)	34 (24.8)	35 (25.5)	
Consumption of cod liver oil or omega-3 fatty acids supplements, *n* (%)				0.254
No	229 (83.6)	111 (81.0)	118 (86.1)	
Yes	45 (16.4)	26 (19.0)	19 (13.9)	
Dietary Vitamin D intake, median (25th75th centiles), µg/ day	5.31 (2.55–10.80)	5.24 (2.65–10.74)	5.64 (2.37–12.23)	0.833
Dietary Calcium intake, median (25th–75th centiles), mg/ day	306.43 (128.63–551.72)	271.01 (125.46–490.07)	320.11 (136.38–605.66)	0.247
Active smoking, *n* (%)				0.146
Never	229 (83.6)	120 (87.6)	109 (79.6)	
Stopped smoking	37 (13.5)	15 (10.9)	22 (16.1)	
Current or in the past one month	8 (2.9)	2 (1.5)	6 (4.4)	
Passive smoking, *n* (%)				0.600
No	190 (69.3)	93 (67.9)	97 (70.8)	
Yes	84 (30.7)	44 (32.1)	40 (29.2)	
Physical activity, *n* (%)				0.822
Inactive (<600 MET-min/week)	29 (10.6)	15 (10.9)	14 (10.2)	
Minimally active (600 to <3000 MET-min/week	101 (36.9)	48 (35.0)	53 (38.7)	
Highly active (≥3000 MET-min/week)	144 (52.6)	74 (54.0)	70 (51.1)	
Sedentary activity, *n* (%)				0.260
≤8 h	101 (36.9)	46 (33.6)	55 (40.1)	
>8 h	173 (63.1)	91 (66.4)	82 (59.9)	
Vitamin D, mean (SD), nmol/L	39.44 (14.71)	38.94 (11.97)	39.94 (17.05)	0.575
Deficiency <25 nmol/L, *n* (%)	41 (15.0)	18 (13.1)	23 (16.8)	0.069
Insufficiency 25 to <50 nmol/L, *n* (%)	174 (63.5)	96 (70.1)	78 (56.9)	
Sufficiency ≥50 nmol/L, *n* (%)	59 (21.5)	23 (16.8)	36 (26.3)	
Total cholesterol, mean (SD), mmol/L	5.04 (0.82)	4.98 (0.82)	5.10 (0.81)	0.223
HDL-cholesterol, mean (SD), mmol/L	1.60 (0.32)	1.58 (0.33)	1.63 (0.32)	0.204
LDL-cholesterol, mean (SD), mmol/L	2.77 (0.71)	2.76 (0.65)	2.79 (0.77)	0.786
Triglyceride, mean (SD), mmol/L	1.44 (0.60)	1.40 (0.54)	1.49 (0.65)	0.203

*p*-values were determined from the independent *t*-test or Mann–Whitney test for continuous variables, and the chi-squared test for the categorical variables. SD, standard deviation. METs, metabolic equivalent of task. HDL-cholesterol, high-density lipoprotein cholesterol. LDL-cholesterol, low-density lipoprotein cholesterol.

**Table 2:** Comparisons of plasma vitamin D, lipid, and glycaemic profiles between trial groups at 24–28 weeks gestation.

Variable	Control (*n =* 119)	Intervention (*n =* 112)	Mean Difference (95% CI)	*p* ^b^	Adjusted Difference (95% CI)	*p* ^c^
Vitamin D, mean (SD), nmol/L	53.46 (16.20)	61.45 (16.74)	8.00 (3.72, 12.27)	<0.001	6.52 (2.74, 10.31)	0.001
Deficiency <25 nmol/L, *n* (%)	3 (2.5)	0 (0.0)	NA	<0.001	NA	NA
Insufficiency 25 to <50 nmol/L, *n* (%)	50 (42.0)	25 (22.3)	NA	NA	NA	NA
Sufficiency ≥50 nmol/L, *n* (%)	66 (55.5)	87 (77.7)	NA	NA	NA	NA
Total cholesterol, mean (SD), mmol/L	6.1 (1.02)	6.13 (1.08)	0.02 (0.25, 0.29)	0.885	0.07 (0.11, 0.24)	0.472
HDL-cholesterol, mean (SD), mmol/L	1.80 (0.36)	1.81 (0.35)	0.01 (0.09, 0.10)	0.773	0.03 (0.03, 0.09)	0.403
LDL-cholesterol, mean (SD), mmol/L	3.30 (0.87)	3.30 (0.95)	0.00 (0.24, 0.24)	>0.950	0.00 (0.19, 0.20)	>0.950
Triglyceride, mean (SD), mmol/L	2.23 (0.82)	2.18 (0.80)	−0.05 (−0.26, −0.16)	0.641	−0.10 (−0.29, −0.08)	0.263
Fasting glucose, mean (SD), mmol/L	4.49 (0.75)	4.43 (0.47)	−0.06 (−0.23, −0.11)	0.480	−0.06 (−0.22, −0.11)	0.517
1 h glucose, mean (SD), mmol/L	8.15 (2.07)	7.95 (1.75)	−0.20 (−0.71, −0.30)	0.427	−0.21 (−0.72, −0.30)	0.422
2 h glucose, mean (SD), mmol/L	6.56 (1.77)	6.69 (1.40)	0.13 (0.29, 0.55)	0.554	0.14 (0.28, 0.56)	0.517
Gestational diabetes, *n* (%) ^a^				0.547		
No	94 (80.3)	84 (77.1)	NA	NA	NA	NA
Yes	23 (19.7)	25 (22.9)	NA	NA	NA	NA

^a^ Five participants declined oral glucose tolerance test (control: two, intervention: three). ^b^ *p*-values were determined from the independent *t*-test for continuous variables and the chi-squared test for categorical variables. ^c^ *p*-values were determined from the analysis of covariance test, adjusting for baseline values and duration of intervention, except for glycaemic measures, which were only adjusted for intervention duration. NA, not applicable.

**Table 3:** Comparisons of maternal and birth outcomes between trial groups at delivery.

Variable	Total (*n =* 227)	Control (*n =* 116)	Intervention (*n =* 111)	*p*
Neonatal birth weight, mean (SD), g	3145.81 (436.83)	3122.63 (434.04)	3170.03 (440.39)	0.415
Low birth weight, *n* (%)				0.751
Yes	24 (10.6)	13 (11.2)	11 (9.9)	
No	203 (89.4)	103 (88.8)	100 (90.1)	
Neonatal birth length, mean (SD), cm	48.48 (2.05)	48.26 (2.15)	48.70 (1.92)	0.103
Neonatal head circumference, mean (SD), cm	33.82 (1.38)	33.71 (1.32)	33.93 (1.43)	0.237
Neonatal status, *n* (%)				0.927
Healthy live birth	211 (93.0)	108 (93.1)	103 (92.8)	
Special care unit admission	16 (7.0)	8 (6.9)	8 (7.2)	
Preterm birth, *n* (%)				0.348
No	214 (94.3)	111 (95.7)	103 (92.8)	
Yes	13 (5.7)	5 (4.3)	8 (7.2)	
Gestational hypertension, *n* (%) ^a^				0.394
No	216 (95.2)	109 (94.0)	107 (96.4)	
Yes	11 (4.8)	7 (6.0)	4 (3.6)	
Gestational weight gain, *n* (%) ^b^				0.307
Adequacy	87 (39.7)	39 (34.8)	48 (44.9)	
Insufficiency	44 (20.1)	25 (22.3)	19 (17.8)	
Excessive	88 (40.2)	48 (42.9)	40 (37.4)	
Mode of delivery, *n* (%)				0.724
Normal vaginal delivery	137 (60.4)	69 (59.5)	68 (61.3)	
Instrumental (forceps/vacuum) vaginal delivery	18 (7.9)	11 (9.5)	7 (6.3)	
Elective caesarean section delivery	27 (11.9)	12 (10.3)	15 (13.5)	
Emergency caesarean section delivery	45 (19.8)	24 (20.7)	21 (18.9)	

*p*-values were determined from the independent *t*-test for continuous variables and chi-squared test for categorical variables. ^a^ The numbers of participants with gestational hypertension and pre-eclampsia are four and seven, respectively. ^b^ Total number is not equal to 227 due to missing data.

**Table 4:** Comparisons of plasma lipid profiles at 24–28 weeks gestation, stratified by plasma 25OHD status.

	Vitamin D (Plasma 25OHD)		
	Deficiency < 25 nmol/L	Insufficiency 25 to <50 nmol/L	Sufficiency ≥ 50 nmol/L
Total cholesterol, mean (SD), mmol/L	5.01 (0.87)	5.04 (0.82)	5.05 (0.78)
HDL-cholesterol, mean (SD), mmol/L	1.50 (0.29)	1.61 (0.33)	1.65 (0.32)
LDL-cholesterol, mean (SD), mmol/L	2.81 (0.81)	2.80 (0.68)	2.67 (0.74)
Triglyceride, mean (SD), mmol/L	1.47 (0.60)	1.39 (0.52)	1.59 (0.78)

## Data Availability

Please contact the corresponding author for more information.

## Supplementary Materials

The following supporting information can be downloaded at https://www.mdpi.com/article/10.3390/nu16010146/s1, Table S1: Comparisons of plasma lipid and glycaemic profiles between groups in subsample women with vitamin D insufficiency at both baseline and 24–28 weeks gestation (*n =* 54); Table S2: Comparisons of plasma vitamin D, lipid, and glycaemic profiles between trial groups at 24–28 weeks gestation, stratified by overweight and obesity status; Table S3: Comparisons of maternal and birth outcomes between trial groups at delivery, stratified by overweight and obesity status; Table S4: Comparisons of plasma vitamin D and lipid levels between groups at 24–28 weeks gestation with ≥ 80% compliance; Table S5: Comparisons of maternal and birth outcomes between trial groups with ≥80% compliance at delivery; Table S6: Adverse events and serious adverse events reported.
