# Supplementary material for: The Effect of Vitamin D Supplementation in Pregnant Women with Overweight and Obesity: A Randomised Controlled Trial
Source: Nutrients. 2023 Dec 31;16(1):146. doi: 10.3390/nu16010146 (PMC10780523; doi:10.3390/nu16010146)
Supplement: Supplementary file 1 [file nutrients-16-00146-s001.zip › nutrients-2766731-supplementary.pdf]

Table S1. Comparisons of plasma lipid and glycaemic profiles between groups in subsample women with vitamin D insufficiency at both baseline and 24-28 weeks gestation (n=54).

Variable	Control (n=37)	Intervention (n=17)	Adjusted difference (95% CI)	p[b]
Total cholesterol, mean (SD), mmol/L	6.28 (1.12)	6.15 (1.33)	-0.05 (-0.47, 0.38)	0.829
HDL-cholesterol, mean (SD), mmol/L	1.82 (0.39)	1.53 (0.23)	-0.17 (-0.30, -0.04)	0.012
LDL-cholesterol, mean (SD), mmol/L	3.41 (0.94)	3.35 (1.43)	-0.15 (-0.60, 0.29)	0.485
Triglyceride, mean (SD), mmol/L	2.39 (0.77)	2.67 (1.03)	0.27 (-0.11, 0.66)	0.164
Fasting glucose, mean (SD), mmol/L	4.46 (0.43)	4.61 (0.61)	0.15 (-0.15, 0.45)	0.321
1-hour glucose, mean (SD), mmol/L	7.91 (1.61)	7.88 (1.86)	-0.03 (-1.07, 1.00)	0.948
2-hour glucose, mean (SD), mmol/L	6.52 (1.14)	6.48 (1.51)	-0.04 (-0.81, 0.73)	0.918
Gestational diabetes, n (%)[a]				0.915
No	28 (77.8)	13 (76.5)	NA	
Yes	8 (22.2)	4 (23.5)	NA	

[a]One participant from the control group declined oral glucose tolerance test.

[b]P-values were determined from the analysis of covariance test, adjusting for baseline values and duration of intervention, except for glycaemic measures which were only adjusted for intervention duration.

NA, Not applicable.

Table S2. Comparisons of plasma Vitamin D, lipid and glycaemic profiles between trial groups at 24-28 weeks gestation, stratified by overweight and obesity status.

	Overweight (25–29.9 kg/m²)				Obese (≥ 30.0 kg/m²)			
**Variable**	**Control (n=72)**	**Intervention (n=61)**	**Adjusted difference (95% CI)**	**p[b]**	**Control (n=47)**	**Intervention (n=51)**	**Adjusted difference (95% CI)**	**p[b]**
Vitamin D, mean (SD), nmol/L	53.59 (17.49)	62.20 (15.80)	6.33 (1.22, 11.43)	0.016	53.26 (14.19)	60.56 (17.92)	7.00 (1.13, 12.87)	0.020
Deficiency <25 nmol/L, n (%)	2 (2.8)	0 (0.0)	NA	NA	1 (2.1)	0 (0.0)	NA	NA
Insufficiency 25 to <50 nmol/L, n (%)	31 (43.1)	11 (18.0)	NA	NA	19 (40.4)	14 (27.5)	NA	NA
Sufficiency ≥50 nmol/L, n (%)	39 (54.2)	50 (82.0)	NA	NA	27 (57.4)	37 (72.5)	NA	NA
Total cholesterol, mean (SD), mmol/L	6.30 (0.96)	6.29 (1.14)	-0.19 (-0.43, -0.07)	0.164	5.81 1.05)	5.94 (0.97)	0.14 (0.10, 0.38)	0.240
HDL-cholesterol, mean (SD), mmol/L	1.82 (0.38)	1.87 (0.35)	0.04 (0.04, 0.13)	0.333	1.77 (0.31)	1.75 (0.34)	-0.01 (-0.09, -0.08)	0.902
LDL-cholesterol, mean (SD), mmol/L	3.45 (0.86)	3.39 (1.00)	-0.07 (-0.33, -0.19)	0.578	3.06 (0.83)	3.20 (0.89)	0.16 (0.13, 0.44)	0.278
Triglyceride, mean (SD), mmol/L	2.26 (0.83)	2.24 (0.88)	-0.12 (-0.37, -0.14)	0.369	2.20 (0.82)	2.12 (0.69)	-0.09 (-0.37, -0.18)	0.508
Fasting glucose, mean (SD), mmol/L	4.36 (0.37)	4.32 (0.37)	-0.04 (-0.17, -0.09)	0.066	4.68 (1.07)	4.56 (0.55)	-0.12 (-0.46, -0.23)	0.505
1-hour glucose, mean (SD), mmol/L	7.88 (1.55)	7.63 (1.49)	-0.27 (-0.81, -0.27)	0.325	8.56 (2.62)	8.33 (1.97)	-0.20 (-1.15, -0.76)	0.687
2-hour glucose, mean (SD), mmol/L	6.37 (1.45)	6.67 (1.30)	0.29 (0.19, 0.78)	0.234	6.85 (2.15)	6.71 (1.53)	-0.07 (-0.83, -0.68)	0.848
Gestational diabetes, n (%)[a]								
No	58 (82.9)	49 (83.1)	NA	NA	36 (76.6)	35 (70.0)	NA	NA
Yes	12 (17.1)	10 (16.9)	NA	NA	11 (23.4)	15 (30.0)	NA	NA

[a]Five participants declined oral glucose tolerance test (Control: two, Intervention: three).

[b]P-values were determined from the analysis of covariance test, adjusting for baseline values and duration of intervention, except for glycaemic measures which were only adjusted for intervention duration.

NA, Not applicable.

Table S3. Comparisons of maternal and birth outcomes between trial groups at delivery, stratified by overweight and obesity status.

	**Overweight (25–29.9 kg/m^2)**			**Obese (≥ 30.0 kg/m^2)**		
**Variable**	**Control (n=69)**	**Intervention (n=60)**	**p**	**Control (n=47)**	**Intervention (n=51)**	**p**
Neonatal birth weight, mean (SD), g	3128.80 (416.74)	3089.70 (450.31)	0.610	3113.57 (462.71)	3264.53 (412.83)	0.091
Low birth weight, n (%)			0.158			0.032
Yes	5 (7.2)	9 (15.0)		8 (17.0)	2 (3.9)	
No	64 (92.8)	51 (85.0)		39 (83.0)	49 (96.1)	
Neonatal birth length, mean (SD), cm	48.39 (1.84)	48.40 (2.07)	>0.950	48.06 (2.55)	49.06 (1.68)	0.024
Neonatal head circumference, mean (SD), cm	33.64 (1.32)	33.76 (1.45)	0.622	33.82 (1.32)	34.13 (1.40)	0.266
Neonatal status, n (%)			0.187			0.102
Healthy live birth	66 (95.7)	53 (88.3)		42 (89.4)	50 (98.0)	
Special care unit admission	3 (4.3)	7 (11.7)		5 (10.6)	1 (2.0)	
Preterm birth, n (%)			0.663			0.717
No	67 (97.1)	57 (95.0)		44 (93.6)	46 (90.2)	
Yes	2 (2.9)	3 (5.0)		3 (6.4)	5 (9.8)	
Gestational hypertension, n (%)[a]						0.423
No	66 (95.7)	58 (96.7)	>0.950	43 (91.5)	49 (96.1)	
Yes	3 (4.3)	2 (3.3)		4 (8.5)	2 (3.9)	
Total gestational weight gain, n (%)[b]			0.219			0.542
Adequacy	24 (36.4)	28 (48.3)		15 (32.6)	20 (40.8)	
Insufficiency	15 (22.7)	7 (12.1)		10 (21.7)	12 (24.5)	
Excessive	27 (40.9)	23 (39.7)		21 (45.7)	17 (34.7)	
Mode of delivery, n (%)			0.776			>0.950
Normal vaginal delivery	41 (59.4)	38 (63.3)		28 (59.6)	30 (59.2)	
Instrumental (forceps/vacuum) vaginal delivery	9 (13.0)	5 (8.3)		2 (4.3)	2 (3.9)	
Elective caesarean section delivery	6 (8.7)	7 (11.7)		6 (12.8)	8 (15.7)	
Emergency caesarean section delivery	13 (18.8)	10 (16.7)		11 (23.4)	11 (21.6)	

P-values were determined from the independent t-test for continuous variables and chi-square test for categorical variables.

[a]The numbers of participants with gestational hypertension and pre-eclampsia are four and seven respectively.

[b]Total number is not equal to 227 due to missing data.

Table S4. Comparisons of plasma vitamin D and lipid levels between groups at 24-28 weeks' gestation with ≥80% compliance.

	**Control (n=119)**	**Intervention (n=112)**	**Mean difference (95% CI)**	**p**	**Adjusted difference (95% CI)[a]**	**P**
Vitamin D, nmol/L	53.46 ± 16.20	61.45 ± 16.74	8.00 (3.72, 12.27)	<0.001	6.52 (2.74, 10.31)	0.001
Severe deficiency <25 nmol/L, n (%)	3 (2.5)	0 (0.0)		<0.001		
Deficiency 25 to <50 nmol/L, n (%)	50 (42.0)	25 (22.3)				
Sufficiency ≥50 nmol/L, n (%)	66 (55.5)	87 (77.7)				
Total cholesterol, mmol/L	6.11 ± 1.02	6.13 ± 1.08	0.02 (0.25, 0.29)	0.885	0.07 (0.11, 0.24)	0.472
HDL-cholesterol, mmol/L	1.80 ± 0.36	1.81 ± 0.35	0.01 (0.09, 0.10)	0.773	0.03 (0.03, 0.09)	0.403
LDL-cholesterol, mmol/L	3.30 ± 0.87	3.30 ± 0.95	0.00 (0.24, 0.24)	>0.950	0.00 (0.19, 0.20)	>0.950
Triglyceride, mmol/L	2.23 ± 0.82	2.18 ± 0.80	-0.05 (-0.26, -0.16)	0.641	-0.10 (-0.29, -0.08)	0.263
Fasting glucose, mmol/L	4.49 ± 0.75	4.43 ± 0.47	-0.06 (-0.23, -0.11)	0.480	-0.06 (-0.22, -0.11)	0.517
1-hour glucose, mmol/L	8.15 ± 2.07	7.95 ± 1.75	-0.20 (-0.71, -0.30)	0.427	-0.21 (-0.72, -0.30)	0.422
2-hour glucose, mmol/L	6.56 ± 1.77	6.69 ± 1.40	0.13 (0.29, 0.55)	0.554	0.14 (0.28, 0.56)	0.517
Gestational diabetes, n (%)[a]				0.525		
No	95 (80.5)	84 (77.1)				
Yes	23 (19.5)	25 (22.9)				

Data are presented as mean ± standard deviation, unless otherwise indicated. P-values were determined from the Student's t-test, ANCOVA or the chi-Square test, where appropriate. Adjusted for baseline values and duration of intervention, except for glycaemic measures which were only adjusted for intervention duration.

[a]Four declined oral glucose tolerance test (Control: two, Intervention: two). One (control) out of four declined was not diagnosed with gestational diabetes at delivery.

Table S5. Comparisons of maternal and birth outcomes between trial groups with ≥80% compliance at delivery.

Variable	Total (n=182)	Control (n=98)	Intervention (n=84)	p
Neonatal birth weight, grams	3159.73 ± 434.69	3116.78 ± 419.51	3209.85 ± 449.07	0.150
Neonatal birth weight, n (%)				0.695
Neonatal birth length, cm	48.50 ± 2.01	48.21 ± 2.04	48.82 ± 1.94	0.042
Neonatal head circumference, cm	33.83 ± 1.33	33.65 ± 1.24	34.05 ± 1.40	0.042
Neonatal status, n (%)				0.782
Healthy live birth	170 (93.4)	92 (93.9)	78 (92.9)	
Special care unit admission	12 (6.6)	6 (6.1)	6 (7.1)	
Gestation age at birth, weeks	38.67 ± 1.18	38.68 ± 1.07	38.66 ± 1.30	0.928
Preterm birth, n (%)				0.230
No	171 (94.0)	94 (95.9)	77 (91.7)	
Yes	11 (6.0)	4 (4.1)	11 (6.0)	
Gestational hypertension, n (%)[a]				0.688
No	172 (94.5)	92 (93.9)	80 (95.2)	
Yes	10 (5.5)	6 (6.1)	4 (4.8)	
Total gestational weight gain, n (%)[b]				0.664
Adequacy	64 (36.4)	32 (33.7)	32 (39.5)	
Insufficiency	39 (22.2)	23 (24.2)	16 (19.8)	
Excessive	73 (41.5)	40 (42.1)	33 (40.7)	
Mode of delivery, n (%)				0.880
Normal vaginal delivery	111 (61.0)	59 (60.2)	52 (61.9)	
Instrumental (forceps/vacuum) vaginal delivery	14 (7.7)	9 (9.2)	5 (6.0)	
Elective caesarean section delivery	23 (12.6)	12 (12.2)	11 (13.1)	
Emergency caesarean section delivery	34 (18.7)	18 (18.4)	16 (19.0)	

Data are presented as mean ± standard deviation, unless otherwise indicated. P-values were determined from the Student's t-test or the chi-Square test, where appropriate.

[a]The numbers of participants with pregnancy-induced hypertension and pre-eclampsia are four and six respectively.

[b]Total number is not equal to 182 due to missing data.

Table S6. Adverse events and serious adverse events reported.

	Total (n=274)	Control (n=137)	Intervention (n=137)
Nausea and vomiting, n (%)			
No	271 (98.9)	136 (99.3)	135 (98.5)
Yes	3 (1.1)	1 (0.7)	2 (1.5)
Migraine, n (%)			
No	273 (99.6)	136 (99.3)	137 (100.0)
Yes	1 (0.4)	1 (0.7)	0 (0.0)
Hospitalisation for blood glucose monitoring, n (%)			
No	273 (99.6)	136 (99.3)	137 (100.0)
Yes	1 (0.4)	1 (0.7)	0 (0.0)
Placenta previa, n (%)			
No	273 (99.6)	136 (99.3)	137 (100.0)
Yes	1 (0.4)	1 (0.7)	0 (0.0)
Hospitalisation for obstetric cholestasis, n (%)			
No	273 (99.6)	136 (99.3)	137 (100.0)
Yes	1 (0.4)	1 (0.7)	0 (0.0)
Hospitalisation for pre-eclampsia, n (%)			
No	273 (99.6)	136 (99.3)	137 (100.0)
Yes	1 (0.4)	1 (0.7)	0 (0.0)
Miscarriage, n (%)			
No	268 (97.8)	134 (97.8)	134 (97.8)
Yes	6 (2.2)	3 (2.2)	3 (2.2)
Termination of pregnancy, n (%)			
No	272 (99.3)	136 (99.3)	136 (99.3)
Yes	2 (0.7)	1 (0.7)	1 (0.7)
